# Supplementary material for: ITA-IMMUNO-PET: The Role of [18F]FDG PET/CT for Assessing Response to Immunotherapy in Patients with Some Solid Tumors
Source: Cancers (Basel). 2023 Jan 31;15(3):878. doi: 10.3390/cancers15030878 (PMC9913289; doi:10.3390/cancers15030878)
Supplement: Supplementary file 1 [file cancers-15-00878-s001.zip › Figure S2.pdf]

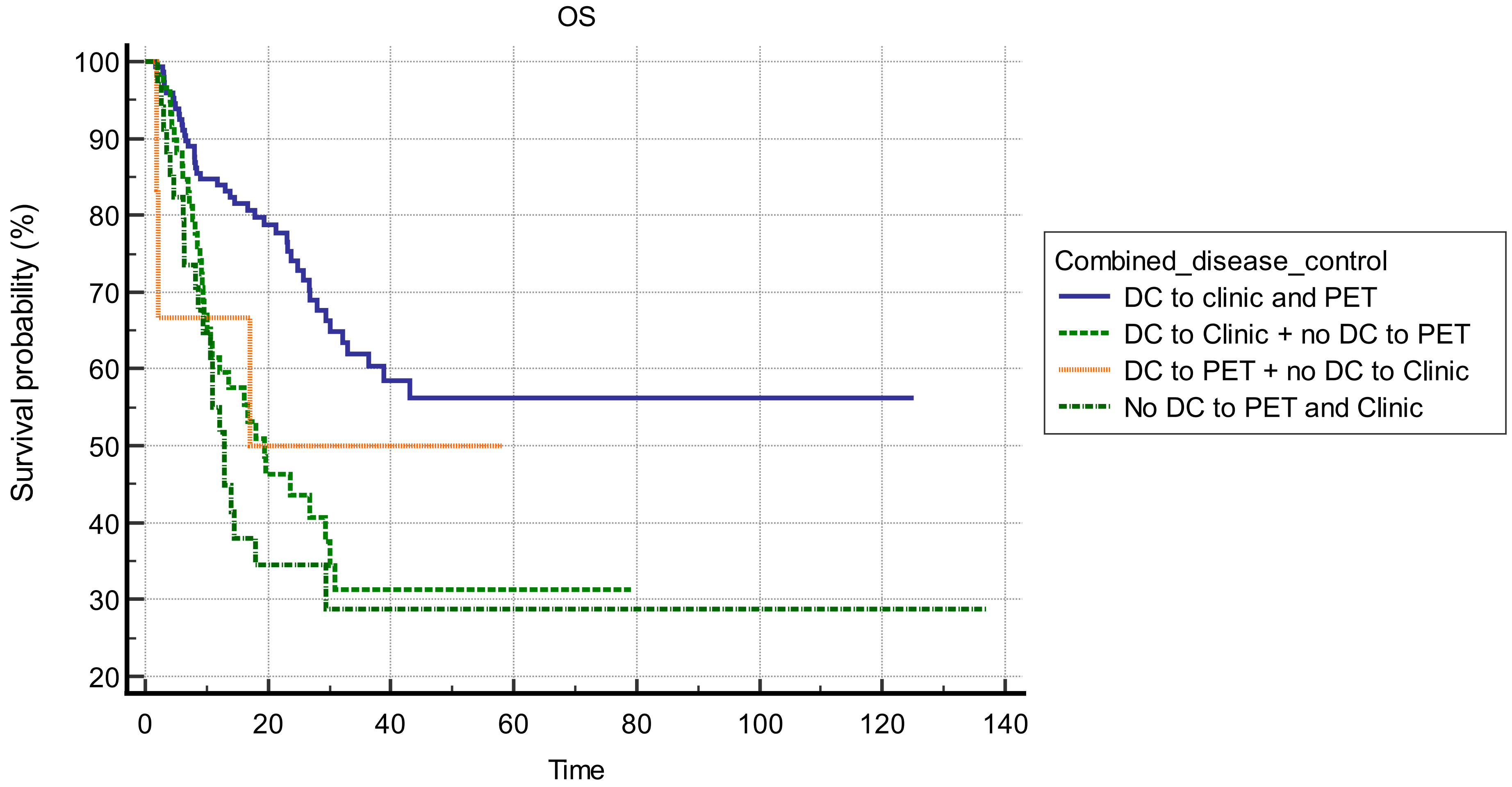

Number at risk

Group: DC to clinic and PET							
148	76	31	12	4	1	1	0
Group: DC to Clinic + no DC to PET							
59	19	7	4	0	0	0	0
Group: DC to PET + no DC to Clinic							
6	3	3	0	0	0	0	0
Group: No DC to PET and Clinic							
34	10	2	1	1	1	1	0

Chi-squared	24,5556
DF	3
Significance	P < 0,0001

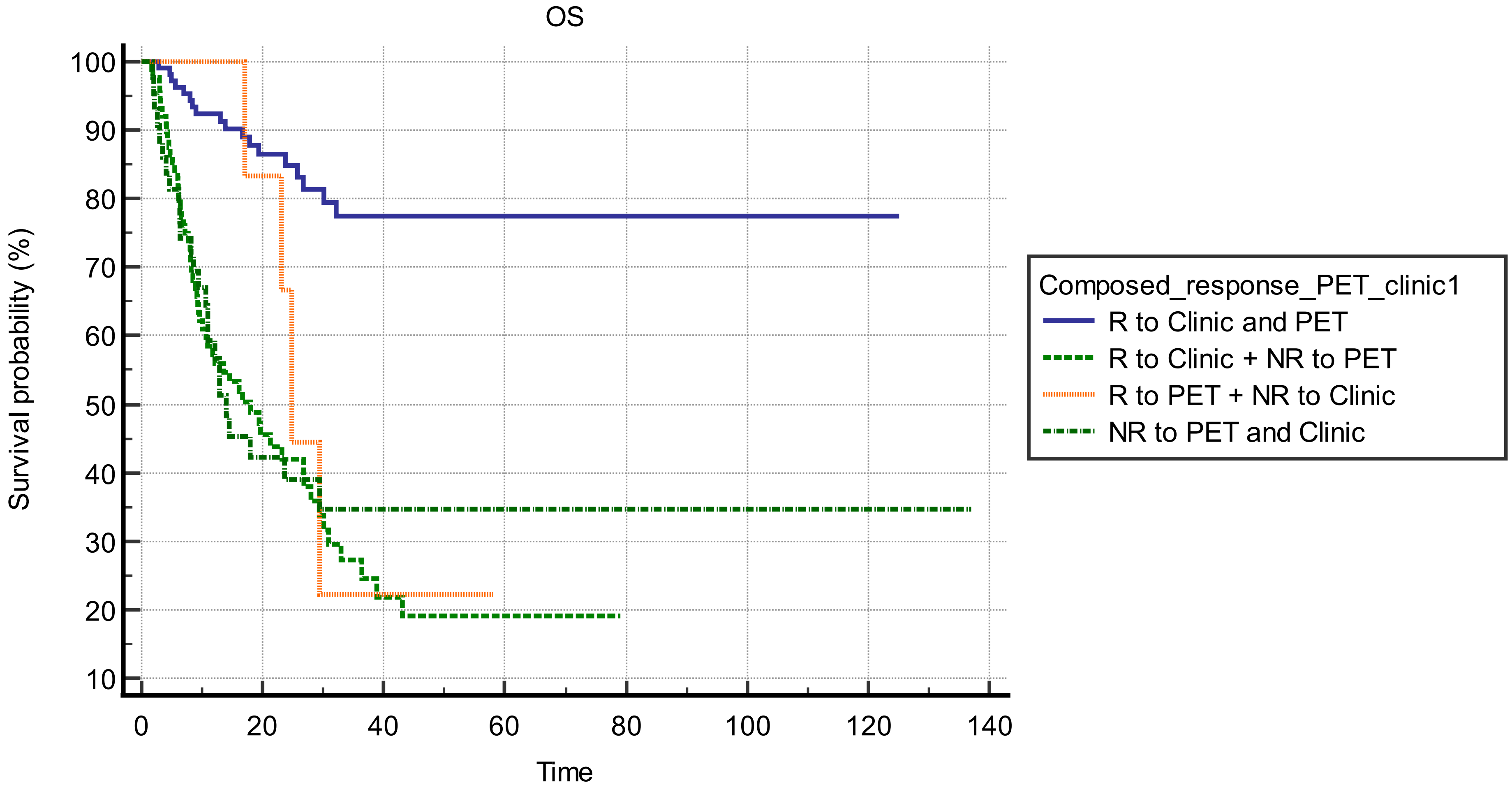

Number at risk

Group	0	20	40	60	80	100	120	140
Group: R to Clinic and PET	109	62	30	12	4	1	1	0
Group: R to Clinic + NR to PET	88	27	8	4	0	0	0	0
Group: R to PET + NR to Clinic	6	5	1	0	0	0	0	0
Group: NR to PET and Clinic	44	14	4	1	1	1	1	0

Chi-squared	54,4202
DF	3
Significance	P < 0,0001
