# Supplementary material for: ITA-IMMUNO-PET: The Role of [18F]FDG PET/CT for Assessing Response to Immunotherapy in Patients with Some Solid Tumors
Source: Cancers (Basel). 2023 Jan 31;15(3):878. doi: 10.3390/cancers15030878 (PMC9913289; doi:10.3390/cancers15030878)
Supplement: Supplementary file 1 [file cancers-15-00878-s001.zip › Supplementary Tables_REV.pdf]

**Table S1.** Involved centers and study design

| | |
|---|---|
| Name of involved centers | IRCCS CROB Referral Cancer Center of Basilicata, Rionero in Vulture (PZ); Azienda Ospedaliero-Universitaria of Parma, Parma; Nuclear Medicine, IRCCS Ospedale Policlinico San Martino, Genova; IRCCS Istituto Romagnolo per lo Studio dei Tumori (IRST), Dino Amadori, Meldola (FC); Humanitas Istituto Clinico Catanese, Misterbianco (CT); Azienda Ospedaliero Universitaria di Ferrara; IRCCS Regina Elena National Cancer Institute, Rome; ASO S.Croce e Carle, Cuneo; Department of Medical Sciences, University of Turin; Veneto Institute Of Oncology IOV - IRCCS, Padova; ICS Maugeri Spa Sb – IRCCS, Pavia; AORN Ospedali dei Colli, Napoli; University of Brescia and ASST Spedali Civili di Brescia; Interdisciplinary Department of Medicine, University of Bari "Aldo Moro", Bari; Az. Ospedaliera Ospedali Riuniti Villa Sofia-Cervello di Palermo; Fondazione IRCCS Istituto Nazionale dei Tumori, Milano; Central Hospital Bolzano (SABES-ASDAA), Bolzano-Bozen |
| Study design | The expected PMR and PMD rates based on FDG PET/CT findings after starting immunotherapy were 21-22% and 15-20%, respectively ([23],[24]), so the observed PMR and PMD rates were set at 0.25 and 0.10, respectively. A sample size of 120, 160, 197 or 276 patients was required for alpha 0.05 and beta values of 80%, 90%, 95% and 99%, respectively). |

**Table S2.** Agreement between serial PET scan and clinical assessment.

| **N of evaluation*** | **N of patients** | **Response to therapy** | | **Disease control** | |
|---|---|---|---|---|---|
| | | **K value** | ***P value*** | **K value** | ***P value*** |
| First (median: 3 mo.) | 252 | 0.27 | < *0.0001* | 0.37 | < *0.0001* |
| Second (median: 6 mo.) | 167 | 0.25 | < *0.0001* | 0.54 | < *0.0001* |
| Third (median: 12 mo.) | 85 | 0.29 | 0.698 | 0.26 | < *0.005* |
| Fourth (median: 16 mo.) | 38 | 0.14 | 0.217 | 0.37 | < *0.005* |
| Fifth (median: 21 mo.) | 20 | - | - | 0.33 | *0.068* |

*data based on the time interval between imaging/clinical assessment and the start of immunotherapy

**Table S3.** Overall Survival based on PET and Clinical response

| | **N pts** | **Mean±SD OS*** | ***P value*** |
|---|---|---|---|
| Response PET1 | | | |
| Responders (CMR+PMR) | 131 | 29±21 | *0.001* |
| No-responders (SMD+PMD) | 145 | 18±17 | |
| Disease control PET1 | | | |
| Disease control (CMR, PMR, SMD) | 184 | 26±20 | *0.001* |
| No disease control (PMD) | 122 | 18±18 | |
| Response Clinical1 | | | |
| Responders (Improvement) | 197 | 25±21 | *0.005* |
| No-responders (stable, worsening) | 50 | 20±21 | |
| Disease control Clinical1 | | | |
| Disease control (stable, improvement) | 207 | 25±20 | *0.201* |
| No disease control (worsening) | 40 | 20±23 | |
| Response PET2 | | | |
| Responders (CMR+PMR) | 75 | 36+25 | *0.001* |
| No-responders (SMD+PMD) | 124 | 22+18 | |
| Disease control PET2 | | | |
| Disease control (CMR, PMR, SMD) | 117 | 32±23 | *0.001* |
| No disease control (PMD) | 82 | 21±19 | |
| Response Clinical2 | | | |
| Responders (Improvement) | 17 | 32+17 | *0.361* |
| No-responders (stable, worsening) | 156 | 23+24 | |
| Disease control Clinical2 | | | |
| Disease control (stable, improvement) | 136 | 31±24 | *0.001* |
| No disease control (worsening) | 37 | 14±9 | |
| Response PET3 | | | |
| Responders (CMR+PMR) | 33 | 47+30 | *0.001* |
| No-responders (SMD+PMD) | 69 | 26+14 | |
| Disease control PET3 | | | |
| Disease control (CMR, PMR, SMD) | 68 | 35+25 | *0.145* |
| No disease control (PMD) | 34 | 28+17 | |
| Response Clinical3 | | | |
| Responders (Improvement) | 7 | 25+6 | *0.348* |
| No-responders (stable, worsening) | 78 | 35+25 | |
| Disease control Clinical3 | | | |

| | | | |
|---|---|---|---|
| Disease control (stable, improvement) | 76 | 35±25 | *0.152* |
| No disease control (worsening) | 9 | 23±12 | |
| Response PET4 | | | |
| Responders (CMR+PMR) | 16 | 39+19 | *0.065* |
| No-responders (SMD+PMD) | 30 | 29+15 | |
| Disease control PET4 | | | |
| Disease control (CMR, PMR, SMD) | 28 | 33±17 | *0.741* |
| No disease control (PMD) | 18 | 31±18 | |
| Response Clinical4 | | | |
| Responders (Improvement) | 3 | 26+12 | *0.433* |
| No-responders (stable, worsening) | 37 | 35+18 | |
| Disease control Clinical4 | | | |
| Disease control (stable, improvement) | 36 | 36±19 | *0.152* |
| No disease control (worsening) | 4 | 22±10 | |
| Response PET5 | | | |
| Responders (CMR+PMR) | 6 | 42+12 | *0.116* |
| No-responders (SMD+PMD) | 17 | 32+12 | |
| Disease control PET5 | | | |
| Disease control (CMR, PMR, SMD) | 14 | 37±12 | *0.381* |
| No disease control (PMD) | 9 | 32±13 | |
| Response Clinical5 | | | |
| Responders (Improvement) | - | - | - |
| No-responders (stable, worsening) | - | - | |
| Disease control Clinical5 | | | |
| Disease control (stable, improvement) | 20 | 34±12 | *0.225* |
| No disease control (worsening) | 2 | 46±22 | |

*expressed in months; CMR=complete metabolic response; PMR= partial metabolic response; SMD=stable metabolic disease; PMD=progressive metabolic disease

**Table S4.** Univariate analysis for the prediction of Overall Survival

| **Variables** | **Univariate analysis** | | |
|---|---|---|---|
| | **HR** | **CI95%** | **P** |
| Type of disease | | | |
| Lung cancer | Ref | Ref | Ref |
| Melanoma | 2.04 | 0.88-4.68 | 0.09 |

| | | | |
|---|---|---|---|
| Other cancer (gynecological, head and neck and breast cancer) | 1.21 | 0.50-2.88 | 0.67 |
| Comorbidity | | | |
| Yes vs. no | 3.72 | 2.32-5.96 | 0.001 |
| Treatments before immunotherapy | | | |
| Yes vs. no | 0.17 | 0.05-0.54 | 0.003 |
| Rate of immunotherapy administration | | | |
| Weekly | Ref | Ref | Ref |
| Two-weekly | - | - | - |
| Three-weekly | 2.63 | 1.35-5.13 | 0.005 |
| Others | 1.19 | 0.59-2.38 | 0.62 |
| Combination of immunotherapy and other treatments | | | |
| Yes vs. no | 2.11 | 1.13-3.94 | 0.018 |
| PET response 1 | | | |
| Non-responders vs. responders | 0.41 | 0.28-0.59 | 0.001 |
| Clinical response 1 | | | |
| Non responders vs. responders | 0.49 | 0.31-0.77 | 0.002 |
| PET response 2 | | | |
| Non responders vs. responders | 0.23 | 0.14-0.39 | 0.001 |
| Clinical response 2 | | | |
| Non responders vs. responders | 0.17 | 0.09-0.28 | 0.001 |
| PET response 3 | | | |
| Non responders vs. responders | 0.053 | 0.01-0.23 | 0.001 |
| Clinical response 3 | | | |
| Non responders vs. responders | 0.19 | 0.06-0.65 | 0.007 |
