# Supplementary figures and images for: ITA-IMMUNO-PET: The Role of [18F]FDG PET/CT for Assessing Response to Immunotherapy in Patients with Some Solid Tumors
Source: Cancers (Basel). 2023 Jan 31;15(3):878. doi: 10.3390/cancers15030878 (PMC9913289; doi:10.3390/cancers15030878)

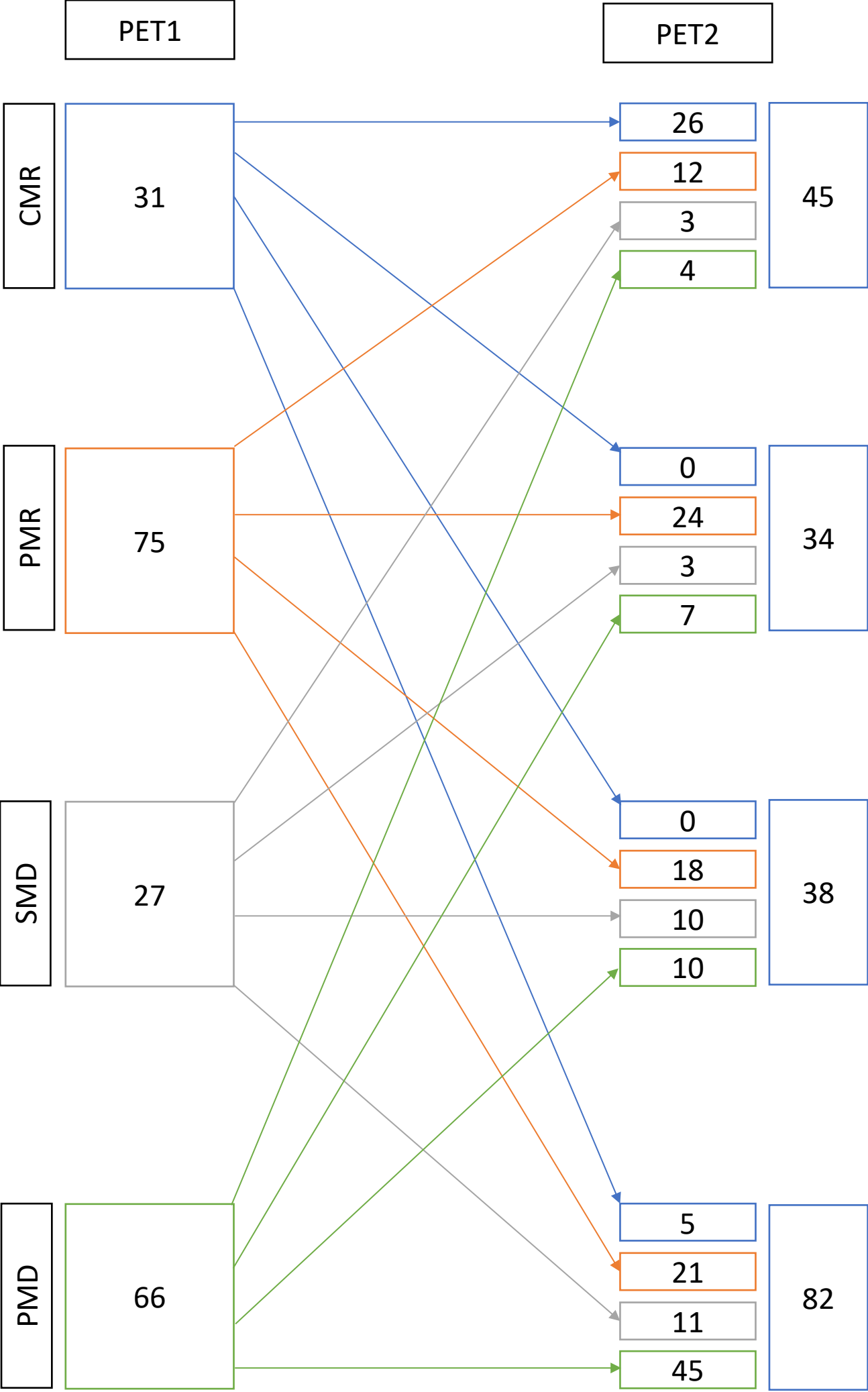

Supplement: Supplementary file 1 [file cancers-15-00878-s001.zip › Figura S1_REV.pdf]
